# Supplementary material for: A substrate-induced gating mechanism is conserved among Gram-positive IgA1 metalloproteases
Source: Commun Biol. 2022 Nov 7;5:1190. doi: 10.1038/s42003-022-04173-3 (PMC9637739; doi:10.1038/s42003-022-04173-3)
Supplement: Supplementary file 2 — Supplementary Information-New [file 42003_2022_4173_MOESM2_ESM.pdf]

Supplementary Information, Redzic et al. (4 Figures, 1 Table)

CLUSTAL O(1.2.4) multiple sequence alignment

```
G.haemolysans(WP_040464465)          MRKYLEEKYNKFSLRKLTVGVCSMTIGSFFLVSTVQPEDYVVKAADNAIVHYKYVGEDNL 60
S.pneumoiae(WP_000417171.1)          MEKYFGEKQERFSFRKLSVGLVSATISSLFFMSVLASS--SVDAQETAGVHYKYVADSEL 58
S.oralis(WP_000713080.1)             MKKFFGEKQHRFSLRKLAIGLVSASISSLFFVSIASSG--TVFAQENVAVHYKYVTDTEL 58
S.Sanguis(WP_002913590.1)            MKKFLGEKQTRFAFRKLAVGLVSAAISSLFFVSIVGVD--SVQAQEKLNVHYKYVTDTEI 58
                                      * *:. **  .*:.***..*. * .* *.*..*       * * :.  ****** : .:
G.haemolysans(WP_040464465)          TDKEKELIKKEVPSVVSSKEETYYLVFKPTKTTQLNKLPNTGLNYGVGSMLLGGM---LG 117
S.pneumoiae(WP_000417171.1)          SSEEKKQLVYDIPTYVENDDETYYLVYKLNSQNQLAELPNTGSKNERQALVAGASLAALG 118
S.oralis(WP_000713080.1)             SGQEKDLIVKDIPKIAEDSESTYYLVYRMDEKAQLGQLPNTGGQNSLTSVLSGGVLASIG 118
S.Sanguis(WP_002913590.1)            TPQEKELIVSGVPRMPEGNEETYYLVYRLNSNAGAKTLPNTGDNNSNTMMAAGLLLTTIG 118
                                      : :**. :   :*    ....:*****:: .       ***** :     :  *     :*
G.haemolysans(WP_040464465)          LVVVVVAKGKNKSRKILSILLVTSLGATTLELPARAMEDLQLSVYNMDYNLKVGDKLPEI 177
S.pneumoiae(WP_000417171.1)          ILIFAVSKKKVKNKTVLHLVLVAGMGNGV-LVSVHALENHLLLNYNTDYELTSGEKLPLP 177
S.oralis(WP_000713080.1)             FLIFVVSKKKGKKKALLKVVLITGMGSGL-ASSVQAIENQLLIQYNQEYQLSQGDSLPLP 177
S.Sanguis(WP_002913590.1)            LVVFAVSKRKVQSKFLLTVLVGASVGGGL-ILSVDALENGSLLQYNAEYQVSAGESLPSP 177
                                      :....*.* * .:. .* ... ..:*       .*.*.  *  ** .*:..  *. **
G.haemolysans(WP_040464465)          SSIPGYSFVGFIKNEAETKKENEEVKEQITSQQHNKKQPELKENTDENVIENKQENKTTL 237
S.pneumoiae(WP_000417171.1)          KEISGYTYIGYIKEGKTTSDFEVSNQEKS------AATPTKQQKVDYNVTPNFVDHPSTV 231
S.oralis(WP_000713080.1)             RALSGYTYLGYIKQDKEINQQ--------------ETAARDQKLDYTVQPHFQANEGGQ 222
S.Sanguis(WP_002913590.1)            GEISGYTYVGYIKDESIKKLLDNKIPDNQQNANVDKEALNQNKKLDYSVS---------- 227
                                       : **:..*.**:    .                ..: * .*
G.haemolysans(WP_040464465)          K--------------------------ISDKK------------EDKKVIE--NINKKD 256
S.pneumoiae(WP_000417171.1)          QAIQEQTPVSSTKPTEVQVVEKPFSTELINPRKEEKQSSDSQEQLAEHKNLETKKEEKIS 291
S.oralis(WP_000713080.1)             KAGDEQKAPSSTSPADKT----------------------------------IPSQDLS 247
S.Sanguis(WP_002913590.1)            -----------------------------------------------------FDKNGL 233
                                                                             :.
G.haemolysans(WP_040464465)          EKKVQGVNTVNPQDEVLAGKLTKPELLYSDKIIETPLKYNQIIESNDQLPEGTTRIKQQG 316
S.pneumoiae(WP_000417171.1)          PKEKTGVNTLNPQDEVLSGQLNKPELLYREETIETKIDFQEEIQENPDLAEGTVRVKQEG 351
S.oralis(WP_000713080.1)             NQKPSGIASVDPQDEVLAGRVNKPELLYKDQEIVTKLDVSEVVQENPELTEGTIHVKQEG 307
S.Sanguis(WP_002913590.1)            KNQTVGVNTIEPQDEVLSGRVAKPELLYKETSIETEIAYGEQIQENPDLAEGTVRVKQEG 293
                                      ::  *: :.:******.*:.: ****** :  * * :   : ::.* .* *** ::**.*
G.haemolysans(WP_040464465)          KEGKKTEVIRMFTVEGKEVSRELISTKTEEPVSEIIEKGTKKAVSNVITKGQKLVKPAVE 376
S.pneumoiae(WP_000417171.1)          KLGKKVEIVRIFSVNKEEVSREIVSTSTTAPSPRIVEKGTKKTQVIKEQPE--------- 402
S.oralis(WP_000713080.1)             RAGKKIELVRIFTVENQEISREVLSTKVEEALPRIVEKGTKKAVVPSEAPQ--------- 358
S.Sanguis(WP_002913590.1)            KPGRKIEVVRIFTVDNAEVSREVLSTKIEEATPKIVEKGTKKLEAPSEKPV--------- 344
                                      : *.* *..*.*.*.  *.***..**.      .*.******
G.haemolysans(WP_040464465)          VKPEYTGVQAGAIVEPVKAEVPKEYTGVQAGAIVEPAKVETPKEYTGVQAGAIVEPAKAE 436
S.pneumoiae(WP_000417171.1)          -----------------------------TGVEH-----------KDVQSGAIVEPAIQP 422
S.oralis(WP_000713080.1)             -----------------------------SAK---------------------------- 361
S.Sanguis(WP_002913590.1)            -----------------------------TSNLVQPEQVAPLPEYTGVQSGAIV------ 369
                                                 :.
G.haemolysans(WP_040464465)          VSKEYTGVQAGTIVEPAKAEVPKEYTGVQAGAIVEPEK-VEPQYGGVTSGALVKPEKIEA 495
S.pneumoiae(WP_000417171.1)          ELPEA---VVSDKGEPEVQPTLPEAVVTDKG-----ETEVQPESPDTVVSDKGEPEQVAP 474
S.oralis(WP_000713080.1)             ------------KGKPETQAPLPEYTGEQAGAVVAPETAEKPEYTGTQAGAVVEPEQVAP 409
S.Sanguis(WP_002913590.1)            --------------EPEQVASLPEYSGTLSGAIVEPEQ-IEPEIGGVQSGAIVEPEQVTP 414
                                    :*      *      *     *   :*:  ..  .   :**::
G.haemolysans(WP_040464465)          PKEYTGVQAGAVVEPAKAEAPKEYRGVQAGAIVEPEKIESPKEYTGVQAGAVVEPAKAEV 555
S.pneumoiae(WP_000417171.1)          LPEYKGNIEQVKPETPVEKTKEQGPEKTEEVPVKP------------------------- 509
S.oralis(WP_000713080.1)             LPEYQGTQAGAIVEPEKVEP-EVGGVQSGALVEPE------------------------- 443
S.Sanguis(WP_002913590.1)            LPEYTGTQAGAVVSPEQVAPLPEYTGTQSGAIVEP------------------------- 449
                                      ** *    . .
G.haemolysans(WP_040464465)          PKEYRGVQAGAIVEPEKIESPKEYTGEQSGAIVEPEKVETTKEYTGIQAGALVEPEKVEA 615
S.pneumoiae(WP_000417171.1)          ---------------TEETPVNPNEGTTEGTSIQEAENPVQPAEESTTNSEKVSPDTSSE 554
S.oralis(WP_000713080.1)             ---------------TSEKPT--YTGEQSGAIVEPEQVPPTPEYRGTQAGAVVAPETAE- 485
S.Sanguis(WP_002913590.1)            ---------------AQVTPLPEYTGVQSGAIVKPAQVTPLPEYTGTQSGAIVEPEQV-- 492
                                      .       *  .*: ::  :       .   . * *:
G.haemolysans(WP_040464465)          PKEYTGVQAGAIVEPEKVEPPKEYTGVQAGAIVEPEKVEAPKEYTGKIEPLKTENPKPTV 675
S.pneumoiae(WP_000417171.1)          N-------------TGEVSSNPSDSTTSVGESNKPEHNDSKNENSEKTV----------- 590
S.oralis(WP_000713080.1)             --------------------KPEYTGTQSGAIVEPETQSSL------------------- 506
S.Sanguis(WP_002913590.1)            ------------------TPSPEYTGVQAGAIVEPEQVASL------------------- 515
                                      . : .. *   :**   :

G.haemolysans(WP_040464465)          ENNNTAEINNVPKNASALLRMNFVKGNQVLSGTGSATFIAPNVLLTVAHNFINNSADNST 735
S.pneumoiae(WP_000417171.1)          --------EEVPVNPNE----------GTVEGTSNQETEKP------------------- 613
S.oralis(WP_000713080.1)             ------------------------------------------------------------
S.Sanguis(WP_002913590.1)            ------------------------------------------------------------


G.haemolysans(WP_040464465)          GEFIGDKSKNTYEWQTPDGQKGSFTSEDIHFYNKKDYPKGFIYDLAVITLPQSTRRQHAN 795
S.pneumoniae_WP_000417171.1          --------------VQP--------------------------------AEETQTNSGK 626
S.oralis(WP_000713080.1)             --------------------------------------------------PEYTGTQAGA 516
S.Sanguis(WP_002913590.1)            --------------------------------------------------PEYTGSQAGA 525
                                                          : *  : .
```

```
G.haemolysans(WP_040464465)            LVENYSKVNVNDKLNVYGYPRGEYAHLKDTTVEIEQKYANNTYGVQYQGGKAGMSGGGIF 855
S.pneumoniae_WP_000417171.1       IANENTG-EVSNKPSDSKPPV-EESNQ----------------PEKNGT---------- 657
S.oralis(WP_000713080.1)        IVE----------PEQVTP-------L-----------------PEYTGN---------- 532
S.Sanguis(WP_002913590.1)        IVE----------PEQVEPPQ-----------------------EYTGN---------- 541
                          :.:          .                     :  *

G.haemolysans(WP_040464465)            NSKGEVIGLHQNGAENRSGGLILSPTQLDWIRSIIKGKEITPNYDALERHKDEKKDDIKE 915
S.pneumoniae_WP_000417171.1       --------------------------------------ATKPEN---S---GN-TTSENG 672
S.oralis(WP_000713080.1)        ---------------------------------TE---QVKPEA---P--TEK-PKEKPK 550
S.Sanguis(WP_002913590.1)        ---------------------------------IE---PAAPEA---ENPTEKAQEPKEQ 562
                                   *:        :

G.haemolysans(WP_040464465)            EKQVDKKLELRNISNVELYTLENNKYRHVSSLSSVPTNPEAYFMKVKSENFKDVMLPVKS 975
S.pneumoiae(WP_000417171.1)       QTEPEKKLELRNVSDIELYSQTNGTYRQHVSLDGIPENTDTYFVKVKSSAFKDVYIPVAS 732
S.oralis(WP_000713080.1)        EKDPEKTLELRNVSDLELYSQTNGTYKQHVSLDGVPSNPDTYFVKVKSSSFKDVYLPVAS 610
S.Sanguis(WP_002913590.1)        KQEPEKNIELRNVSDVELYSLADGKYKQHVSLDAIPSNQENYFVKVKSSKFKDVFLPISS 622
                          : : .* .****.*.:***.  . *.:  **  .* * . **.****  **** .*. *

                                                    ←--- linker →
G.haemolysans(WP_040464465)            IESARKDNQDVYKIVGQANDLIQHENNITLENYTYYLPKTVNSENGVYTSFKNLVDAMNI 1035
S.pneumoiae(WP_000417171.1)       ITEEKRNGQSVYKITAKAEKLQQELENKYVDNFTFYLDKKAKEENTNFTSFSNLVKAINQ 792
S.oralis(WP_000713080.1)        ITAETKDGQPVYKITAKAEKLQQELENKYVDNFTFYLAKKAREETTTFTSFSNLVKAINQ 670
S.Sanguis(WP_002913590.1)        IVDSTKDGQPVYKITASAEKLKQDVNNKYEDNFTFYLAKKAEREVTNFTSFSNLVQAINN 682
                          *    :.:* ****  .*. * *  .*    .*:*.** *  .  *    .*** *** *.*

G.haemolysans(WP_040464465)            NPYGTFRLGATMDAREVELSDGQESYINKEFSGKLIGENKGKYYAIYNLKKPLFKALSHA 1095
S.pneumoiae(WP_000417171.1)       NPSGTYHLAASLNANEVELGPDERSYIKDTFTGRLIGEKDGKNYAIYNLKKPLFENLSGA 852
S.oralis(WP_000713080.1)        NLSGTYHLAASLNANEVELEPEAKSYIKGTFTGQLIGEKDGKQYAIYNLKKPLFETLSGA 730
S.Sanguis(WP_002913590.1)        NLNGTYYLAASLNANEVELENGASSYIKGRFTGKLFGSKDGKNYAIYNLKKPLFDTLSAA 742
                          *  **: *.*:..*.****      ***:  *:*.*.* .** *********** . ** *

G.haemolysans(WP_040464465)            TIQDLSIKEANVSSKEDAATIAKEAKNDTTIANVHSSGVIAGERSIGGLISQVTDSTISN 1155
S.pneumoiae(WP_000417171.1)       TVEKLSLKNVAISGKNDIGSLANEATNGTKIKQVHVDGVLAGERGVGGLLAKADQSSIAE 912
S.oralis(WP_000713080.1)        RVEKLSLKNVSISGKDDIGSLTYEAQNGTKIKQVHVDGVLAGERGIGGLLAKADQSSITE 790
S.Sanguis(WP_002913590.1)        TVENLTLKDVNISGKTDIGALANEANNATRINNVHVDGVLAGERGIGGLVWKADNSKISN 802
                          ::.*::*:. .*.*  .::. ** * *  **.****.****: .:. :* *.:

G.haemolysans(WP_040464465)            SSFTGRITNTYDTTATYQIGGLVGKLSGVGALIEKSISSIDMATNANTGDQVVGGVAGVV 1215
S.pneumoiae(WP_000417171.1)       SSFKGRIVNTYETTDAYNIGGLVGHLTGKNASIAKSKATVTISSNTNRSDQTVGGLAGLV 972
S.oralis(WP_000713080.1)        SSFKGRIINTYETTAAYNIGGLVGHLTGNKALLTKSKATVAISSNTNSSDQTVGGLAGLV 850
S.Sanguis(WP_002913590.1)        SSFKGRIVNSYETKAPYNIGGLVGQLTGINALVDKSKATITISSNADSTNQTVGGLAGLV 862
                          ***.*** *.*.*    *:*******.*.*  * : ** ::: :.:*.* .:* .***.**:*

G.haemolysans(WP_040464465)            DKKATIRNSYVEGNLNNVKPFGKVGGVVGNLWDRETSEVSNSGNLTNVLSDVNVTNGNAI 1275
S.pneumoiae(WP_000417171.1)       DQDAHIQNSYAEGDINNVKHFGKVAGVAGYLWDRTSGEEKHAGELTNVLSDVNVTNGNAI 1032
S.oralis(WP_000713080.1)        DQDAQIQDSYAEGDINNVKHFGRVAGVAGYLWDRTSNLEKHAGSLTNVLSDVNVTNGNAI 910
S.Sanguis(WP_002913590.1)        EKDALISNSYAEGNINNVKRFGSVAGVAGYLWDRDSSEERHAGRLHNVLSDINVMNGNAI 922
                          :: * * .** **.:****  ** *.**.* ****  .     :.*  * *****.** *****

                                       ←------------------------ linker---------
G.haemolysans(WP_040464465)            AGYDFNGIKATNTYSNKNNKVVKVVQVDDEVLSKDSEEQRGTVLENNIVLEKKIELVPKK 1335
S.pneumoiae(WP_000417171.1)       TGYHYTGMKVANTFSSKANRVFNVTLEKDEVVSKESFEERGTMLDASQIVSKKAEINPLT 1092
S.oralis(WP_000713080.1)        TGYHYNDMKVKDTFSSKANRVYNVTLVKDEVVSKESFEERGTMLDTSQIESKKAAINPLT 970
S.Sanguis(WP_002913590.1)        SGYHYRGMRITDSYSNKDNRVYKVTLEKDEVVTKESLEERGTILDVSQIASKKSEINSLS 982
                          :**. :   :.:* *.* .*  .  ***:.*:* *:***.*: .   :  **  :  .

                                   linker-------------------→
G.haemolysans(WP_040464465)            NTKIEDFNFSSRYETDYKNLKDADVSRLRVYKNIEKLLPFYNRETIVKYGNLVDANNTLY 1395
S.pneumoiae(WP_000417171.1)       LPTVEPLSTSGKKDSDFSKIAHYQANRALVYKNIEKLLPFYNKSTIVKYGNLVKENSLLY 1152
S.oralis(WP_000713080.1)        LPTVEPLSTSGKKDSDFSKVAHYQAKRALAYKNIEKLLPFYNKATIVKYGNLVNENSLLY 1030
S.Sanguis(WP_002913590.1)        APKVETLLTSTNKESDFSKVKDYQASRALAYKNIEKLLPFYNKATIVKYGNLVKEDSTLY 1042
                           .:* :  *  . ..:*.:.: . ...*  .   *************. *********  . : **

G.haemolysans(WP_040464465)            TKDLVSVVPMKDKEVISDINKNKTSINKLLLHYSDNTSQTLDIKYLQDFSKVAEYEIANT 1455
S.pneumoiae(WP_000417171.1)       QKELLSAVMMKDDQVITDIVSNKQTANKLLLHYNDHSSEKFDLKYQTDFANLAEYNLGNT 1212
S.oralis(WP_000713080.1)        QKEVLSAVMMKDNQVITDIVSNKQTANKLLLHYKDHSSEKIDLQYQADFAKLAEYSLGDT 1090
S.Sanguis(WP_002913590.1)        EKEILSAVMMKDNEVITDIASHKEAANKLLIHYKDHSSEKLDLTYQSDFSKLAEYRVGDT 1102
                           *:..* * *** .:**.**  .*  . ****.**.*.:*. :*: *  **:.:.*. *  **...*** . :*

G.haemolysans(WP_040464465)            KLIYTPNTLLHSYNNIVKAVLNDLKSVQYDSDAVRKVLDISSNIKLTELYLDEQFTKTKA 1515
S.pneumoiae(WP_000417171.1)       GLLYTPNQFLYDRDSIVKEVLPELQKLDYQSDAIRKTLGISPEVKLTELYLEDQFSKTKQ 1272
S.oralis(WP_000713080.1)        GLLYTPNQFLYDQSSIIKQVLPDLQNVDYHSEGIRKTLGISPNVKQTELYLEDQFAKTKE 1150
S.Sanguis(WP_002913590.1)        GLIYTPNQFLQNHSSIVNEVLPDLKAVDYQSEAIRNTLGISSGVSLTELYLEEQFAKTKE 1162
                           *.**** .*   . ..:*.: **  .*. :* .:* ..:* *.  ..:. * * **  :  *****.:**.***

G.haemolysans(WP_040464465)            NIEDSLSKLLSADAVIAENSNSIIDNYVIEKIKNNKEALLLGLTYLERWYNFKYDNTSAK 1575
S.pneumoiae(WP_000417171.1)       NLGDSLKKLLSADAGLAS-DNSVTRGYLVDKIKNNKEGLLLGLTYLERWYNFNYGQVNVK 1331
S.oralis(WP_000713080.1)        HLEDSLKKLLSADAGLAG-DNPVTKGYLVDKIKRNKEGLLLGLTYLERWYNFSYGQVNVK 1209
S.Sanguis(WP_002913590.1)        NLANTLEKLLSADAVIAS-ENQTINGYVVDKIKRNKEALLLGLTYLERWYNFNYGDVNVK 1221
                          :: ::*.*******  .*  .*    *:..*** .*** ***************  *.  .. ....  *

G.haemolysans(WP_040464465)            DLVLYHLDFFGKSNSSALDNVIELGKSGFNNLLAKNNVITYNVLLSKNYGTEGLFKALEG 1635
```

```
S.pneumoiae(WP_000417171.1)      DLVMYHPDFFGKGNTSPLDTLIELGKSGFNNLLAKNNVDTYGISLASQHGATDLFSTLEH 1391
S.oralis(WP_000713080.1)         DLVLYHLDFFGKGNASPLDTLIELGKSGFNNLLAKNNVDTYSISLASHHGTTDLFSTLEN 1269
S.Sanguis(WP_002913590.1)        DLVMYHMDFFGKGNVSPLDTIIELGKSGFNNLLAKNNVDAYNISLANNNATKDLFSTLAN 1281
                                 ***.** *****.* * **.:***************** .*.:.*:.:.:  **.:*

G.haemolysans(WP_040464465)      YRKVFLPNVSNNDWFKTQSKAYIVEEKSTIPEVSSKQSKQGTEHSIGVYDRLTSPSWKYQ 1695
S.pneumoiae(WP_000417171.1)      YRKVFLPNTSNNDWFKSETKAYIVEEKSTIEEVKTKQGLAGTKYSIGVYDRITSATWKYR 1451
S.oralis(WP_000713080.1)         YRKVFLPTTSNNDWFKKQTKAYIVEEKSNIEEVKTKQGLAGTKYSISVYDRITSATWKYR 1329
S.Sanguis(WP_002913590.1)        YREVFLPNKTNNQWFKEQTKAYIVEEKSAIDEVRVKQEQAGSKYSIGVYDRITSDTWKYR 1341
                                 **:****..:**:***.:.:*********.* **  **   *:..**.****:**.:***:

G.haemolysans(WP_040464465)      SMVLPLLTLPEEKMIFMIANISTIGFGAYDRYRSSEYPKGDKLNRFVEENAQAAAKRFRD 1755
S.pneumoiae(WP_000417171.1)      NMVLPLLTLPER-SVFVISTMSSLGFGAYDRYRSSDHKAGKALNDFVEENARETAKRQRD 1510
S.oralis(WP_000713080.1)         NMVLPLLTLPEK-SVFVISTMSSLGFGAYDRYRNSDHKAGKALNDFVEENARETAKRQRD 1388
S.Sanguis(WP_002913590.1)        NMVLPLLTMPER-SVFVISTISSLGFGAYDRYRNNEHRAGAELNKFVEDNAQETAKRQRD 1400
                                 .*******:**. .*:*:.:.*:.:*********.:..  *  ** ***:**:.:*** **

G.haemolysans(WP_040464465)      HYDYWYKILDKENKEKLFRSVLVYDAFRFGNDTN--KETQEANFETNNPVIKNFFGPAGN 1813
S.pneumoiae(WP_000417171.1)      HYDYWYRILDEQSREKLYRTILLYDAYKFGDDTTSGKATAEAKFDSSNPAMKNFFGPVGN 1570
S.oralis(WP_000713080.1)         HYDYWYRILDNEGREKLYRTILLYDAYKFGDDTTSGKATLEAKFDSSNPAMKNFFGPVGN 1448
S.Sanguis(WP_002913590.1)        HYDYWYRILDEQGREKLYRNILVYDAYKFGDDTTVDKATVEAQFDSSNPAMKYFFGPVGN 1460
                                 ******:***:....:***.*:.*:***:.**:.*   * * **:*:.:.**:.*:**** **

G.haemolysans(WP_040464465)      NVVHNKHGAYATGDAFYYMAYRMLDKSGAVTYTHEMTHNSDREIYLGGYGRRSGLGPEFY 1873
S.pneumoiae(WP_000417171.1)      KVVHNQHGAYATGDGVYYMSYRMLDKDGAITYTHEMTHDSDQDIYLGGYGRRNGLGPEFF 1630
S.oralis(WP_000713080.1)         KVVHNQHGAYATGDGVYYMSYRMLDKDGAITYTHEMTHDSDQDIYLGGYGRRSGLGPEFF 1508
S.Sanguis(WP_002913590.1)        KVVHNKHGAYATGDSVYYMGYRMLDKDGAITYTHEMTHDSDNEIYLGGYGRRSGLGPEFF 1520
                                 :****:********..***.******.**:*********:**.:********* ******:

G.haemolysans(WP_040464465)      AKGLLQAPDHSYDPTITINSVLKYDDS--ENSTRLQIADPTQRFTNVEDLHNYMHNMFDL 1931
S.pneumoiae(WP_000417171.1)      AKGLLQAPDQPSDATITINSILKHSKSDSTEGSRLQVLDPTERFQNAADLQNYVHNMFDL 1690
S.oralis(WP_000713080.1)         AKGLLQAPDQPSDATITINSILRHSKSDSTEGSRLQILDPTERFQNATDLQNYVHNMFDL 1568
S.Sanguis(WP_002913590.1)        AKGLLQAPDHPDDATITVNSILKYDKNDASEKSRLQVLDPTKRFQNADDLKNYVHNMFDV 1580
                                 *********:. * ***:**:*:.:.....   :.:***:.***:** *  **:**:*****:

G.haemolysans(WP_040464465)      IYTLEILEGRAV-AKLDYNEKNDLLRKIENIYKKDP-DGNSVYATNAVRRLTSDEIKNLT 1989
S.pneumoiae(WP_000417171.1)      IYMMEYLEGQSIVNKLSVYQKMAALRKIENKYVKDPADGNEVYATNVVKELTEAEARNLN 1750
S.oralis(WP_000713080.1)         IYMLEYLEGQSIVNKLNVYQKMAVLRKIENKYVKDPVDGNEVYATNVVKELTEAEAQKLT 1628
S.Sanguis(WP_002913590.1)        IYMLEYLEGMSIVNRLSDVQKVNALRKIENKYVRD-ADGNDVYATNVIKNITMADAQKLN 1639
                                 ** :* ***.:.:  .:*:  .:*   ****** *.:*  ***:*****.:....*  :.:.*.
\
G.haemolysans(WP_040464465)      SFDKLIENDVITRRGYIDQGEYERNGYHTINLFSPIYSALSSKIGTPGDLMGRRMAFELL 2049
S.pneumoiae(WP_000417171.1)      SFESLIDHNILSAREYQ-SGDYERNGYYTIKLFAPIYSALSSEKGTPGDLMGRRIAYELL 1809
S.oralis(WP_000713080.1)         NFDSLIDHNILSAREYQ-SGDYERNGYYTIKLFAPIFSALSSEKGTPGDLMGRRIAYELL 1687
S.Sanguis(WP_002913590.1)        SFNSLIENDILSAREYK-NGDVERNGYHTIKLFSPIYSALSSEKGTPGDLMGRRIAYELL 1698
                                 .*:.:**:......* *  .*:.*****:**:**:**:*****:.**********.*.:***

G.haemolysans(WP_040464465)      AAKGYKEGMVPYISNQYEKEAKDRGSKIRSYGKEIGLVTDDLVLEKVFNKKYGSWVEFKK 2109
S.pneumoiae(WP_000417171.1)      AAKGFKDGMVPYISNQYEEDAKQQGQTINLYGKERGLVTDELVLKKVFDGKYKTWAEFKT 1869
S.oralis(WP_000713080.1)         AAKGFKDGMVPYISNQYEEAAKQKGKTINLYGKERGLVTDELVLDKVFEGKYASWAAFKK 1747
S.Sanguis(WP_002913590.1)        AAKGFKDGMVPYISNQYEDDAKQNGKTISIYGKTRGLVTDDLVLRKVFNGQFNNWTEFKK 1758
                                 ****:*:***********.**:.*.:*  ***  *****:*** ***:.:..:.*.:**.

G.haemolysans(WP_040464465)      DMYKERVEQFSKLNRVSFFDPNGPWGRQKNVTVNNISVLEKMIETAVREDAEDF---TAQ 2166
S.pneumoiae(WP_000417171.1)      AMYQERVDQFGNLKQVTFKDPTKPWPSYGTKTINNVDELQALMDQAVLKDAEG--PRWSN 1927
S.oralis(WP_000713080.1)         AMYKERVDQFKNLKQVTFKDPTKPWPSYATKTINSVTELQELMDQAVLQDAVA--PRWSD 1805
S.Sanguis(WP_002913590.1)        AMYEERKNKFDSLNKVTFDDTRQPWTSYATKTISTVEELQTLMDEAVLQDANDNWYSWSG 1818
                                 **:** ..:* *..*.* *   **     .*:...  *:.... ** :**       :

G.haemolysans(WP_040464465)      VYPDTNSRVLKLKKAIFKAYLDQTKDFRTSIFGGK- 2201
S.pneumoiae(WP_000417171.1)      YDPEIDSAVHKLKRAIFKAYLDQTNDFRSSIFENKK 1963
S.oralis(WP_000713080.1)         YKPEIDSAVHKLKRAIFKAYLDQTKDFRTSIFKK-- 1839
S.Sanguis(WP_002913590.1)        YKPEYNSAVHKLKKAVFKAYLDQTKDFRKSIFENQK 1854
                                 *:.:* * ***:*:********:***.:***
```

Supplementary Figure 1. Sequence comparison between four IgA1Ps. Specific regions are color-coded to match structural figures. These include the conserved LPXTG motif and the G5 domain (gray), the IgA1P NTD1 domain (yellow), the IgA1P NTD[2] domain (red), the IgA1P MD domain (green), the IgA1P CTD domain (purple), and the GhTrp (brown). Sequences were aligned with Clustal Omega through uniprot.org.

```
G.haemolysans(WP_040464465)       GIQAGALVEPEKVEAPKEYTGVQAGAIVEPEKVEPPKEYTGVQAGAIVEPEKVEAPKEYT 660
1agj(WP_001065781.1)  --------------------EVSAEEIK--KHEEKWNKYYGVNA--------FNLPKELF 30
                                  *.*  *   :: *  ::* **:*         .: ***

G.haemolysans(WP_040464465)       GKIEPLKTENPKPTVENNNTAEINNVPKNASALLRMNFVKGNQVLSGTGSATFIAPNVLL 720
1agj(WP_001065781.1)  SKVDEK---------------DRQKYPY---NTIGNVFVKGQTSATG----VLIGKNTVL 68
                      .*::               : :: *      :   ****:   :*     ..*. *.:*

G.haemolysans(WP_040464465)       TVAHNFINNSADNSTGEFIGDKSKNTYEWQTPDGQKGSFTSEDIHFYNKKDYPKGFIYDL 780
1agj(WP_001065781.1)  TNRHIAKFANGDPSKVSFRP--SINTDDNGNTETPYGEYEVKE-----ILQEPFGAGVDL 121
                      *  *     ..* *. .*    * ** :  . :   *.:  ::       : * *    **

G.haemolysans(WP_040464465)       AVITLPQSTRRQ------HANLVENYSKVNVNDKLNVYGYPRGEYAHLKDTTVEIEQKYA 834
1agj(WP_001065781.1)  ALIRLKPDQNGVSLGDKISPAKIGTSNDLKDGDKLELIGYPFDHKVNQMH---RSEIELT 178
                      *:* *   . .            : . ..:: . ***:: ***  .. .:  .   . * : :

G.haemolysans(WP_040464465)       NNTYGVQYQG-GKAGMSGGGIFNSKGEVIGLHQNGAENRSGG--LILSPTQLDWIRSIIK 891
1agj(WP_001065781.1)  TLSRGLRYYGFTVPGNSGSGIFNSNGELVGIHSSKVSHLDREHQINYGVGIGNYVKRIIN 238
                      . : *:.* *    * ** .*****:**:.*:*.. ..: .    :  .    .... **:

G.haemolysans(WP_040464465)       GKEITPNYDALERHKDEKKDDIKEEKQVDKKLELRNISNVELYTLENNKYRHVSSLSSVP 951
1agj(WP_001065781.1)  EKNE-------------------------------------------------------- 242
```

Supplementary Figure 2. Sequence comparison between trypsin-like domains. Sequence alignment between the *G. haemolysans* GhTrp domain (brown) and closest solved structure of *Staphylococcus aureus* epidermolytic toxin A (ETA, PDB entry 1agj). The conserved catalytic triad is highlighted (yellow). Sequences were aligned with Clustal Omega through uniprot.org.

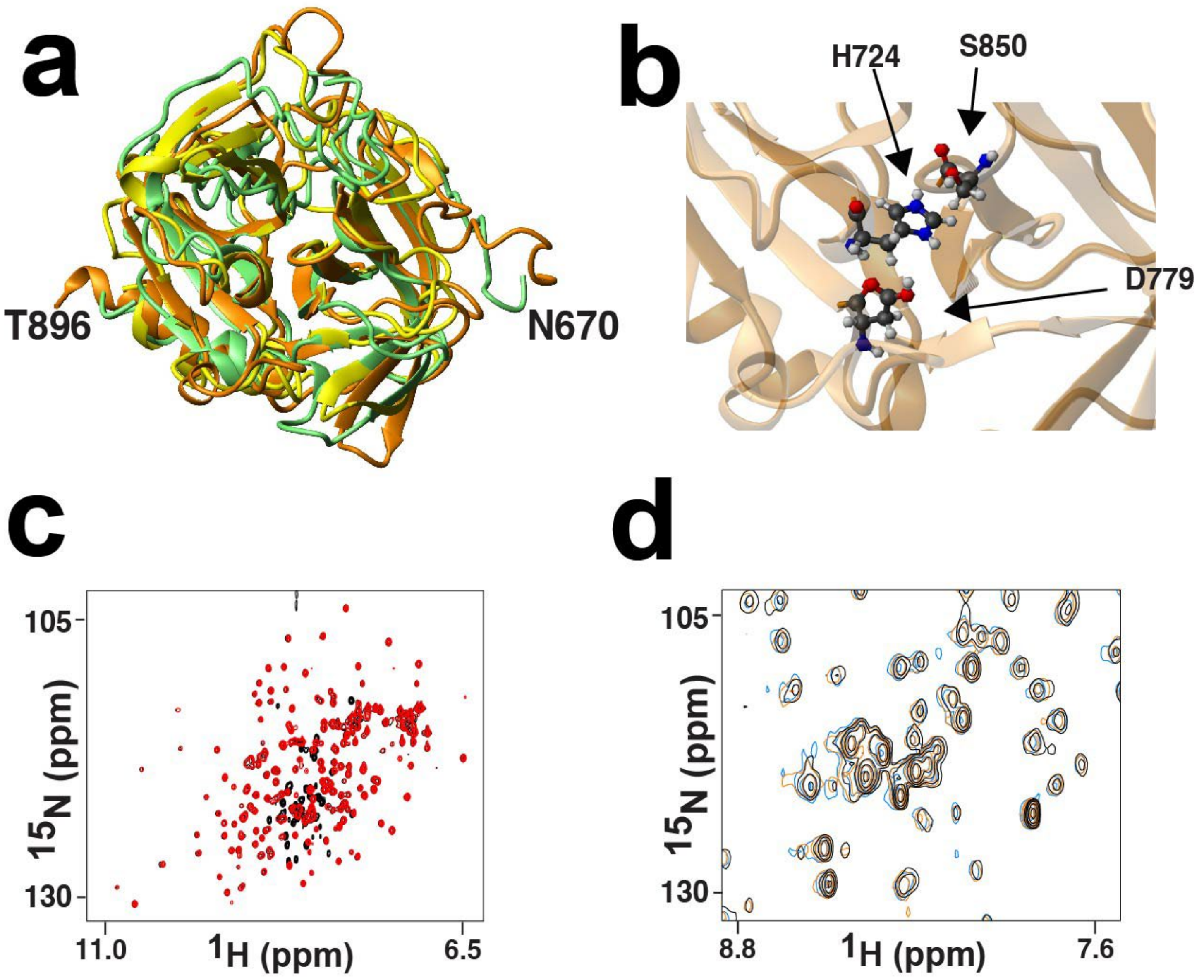

Supplementary Figure 3. Structural interrogation of the *G. haemolysans trypsin-like domain, GhTrp. a) Overlay of three independently determined models of GhTrp that included SWISS-MODEL* (yellow), I-TASSER model (green), and Rosetta model (orange). b) The catalytic triad of *H724, D779, and S850 is shown within the Rosetta model. c)* ^{15}N-HSQC spectra of GhTrp residues 684-896 (red) is shown along with the overlay of the N-terminally extended *construct of GhTrp residues 651-896 (black). d)* ^{15}*N-HSQC spectra of GhTrp residues 651-891 alone (black) and with 50* μ*M (blue) and 100* μ*M (orange) unlabelled G. haemolysans IgA1P region (residues 907-2201).*

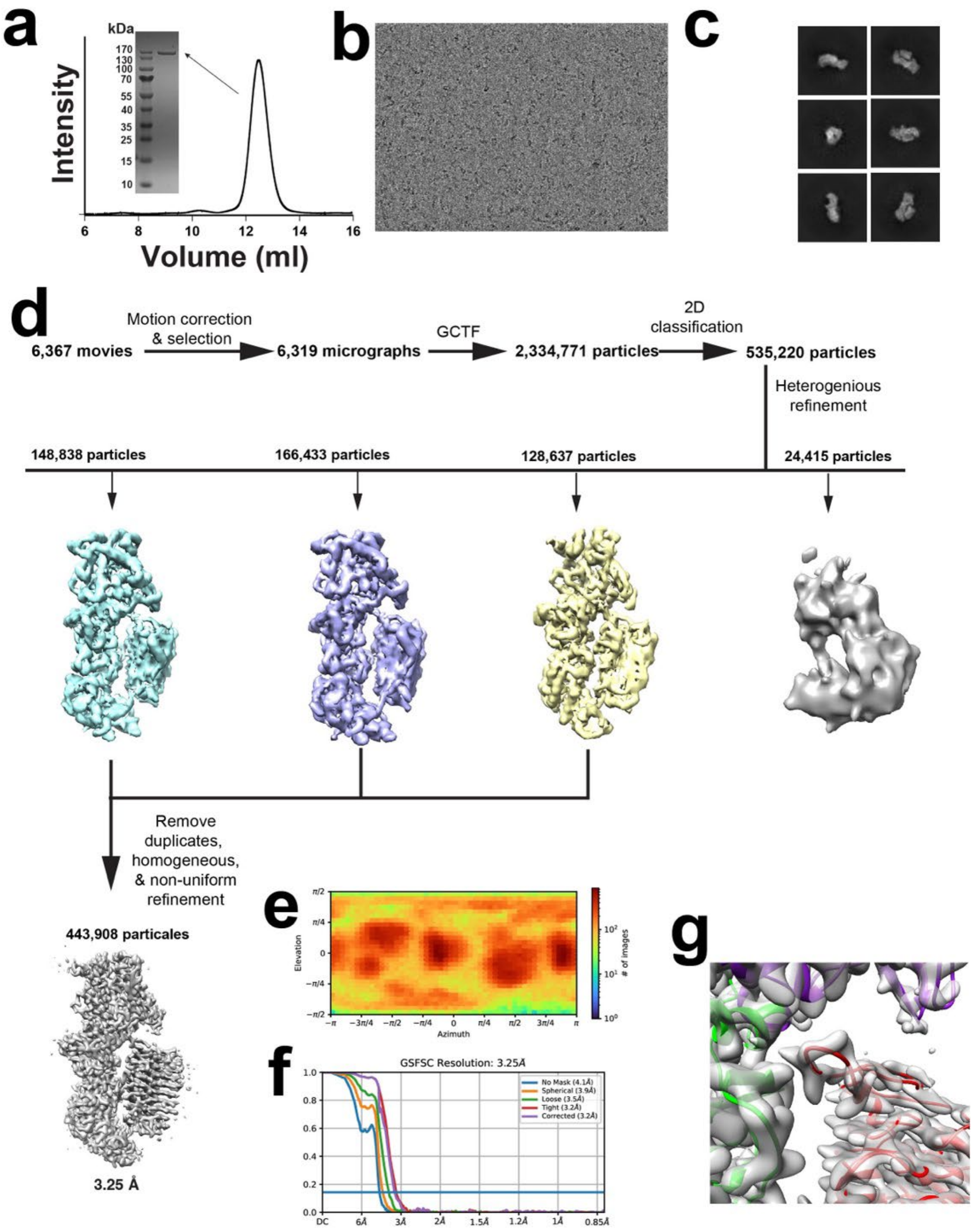

Supplementary Figure 4. Purification and workflow for cryo-EM data processing for substrate free *G. haemolysans* IgA1P. a) Analytical size exclusion chromatography to assess purity of substrate-free *G. haemolysans* IgA1P residues 907-2201 together with an SDS-PAGE gel analysis of this single elution peak. b) Raw cryo-EM micrograph c) Representative 2D class averages of substrate-free *G. haemolysans* IgA1P residues 907-2201. d) EM data processing workflow. e) Angular distribution of the final reconstruction. f) Local resolution map of the final density. g) Density and corresponding model for the active site.

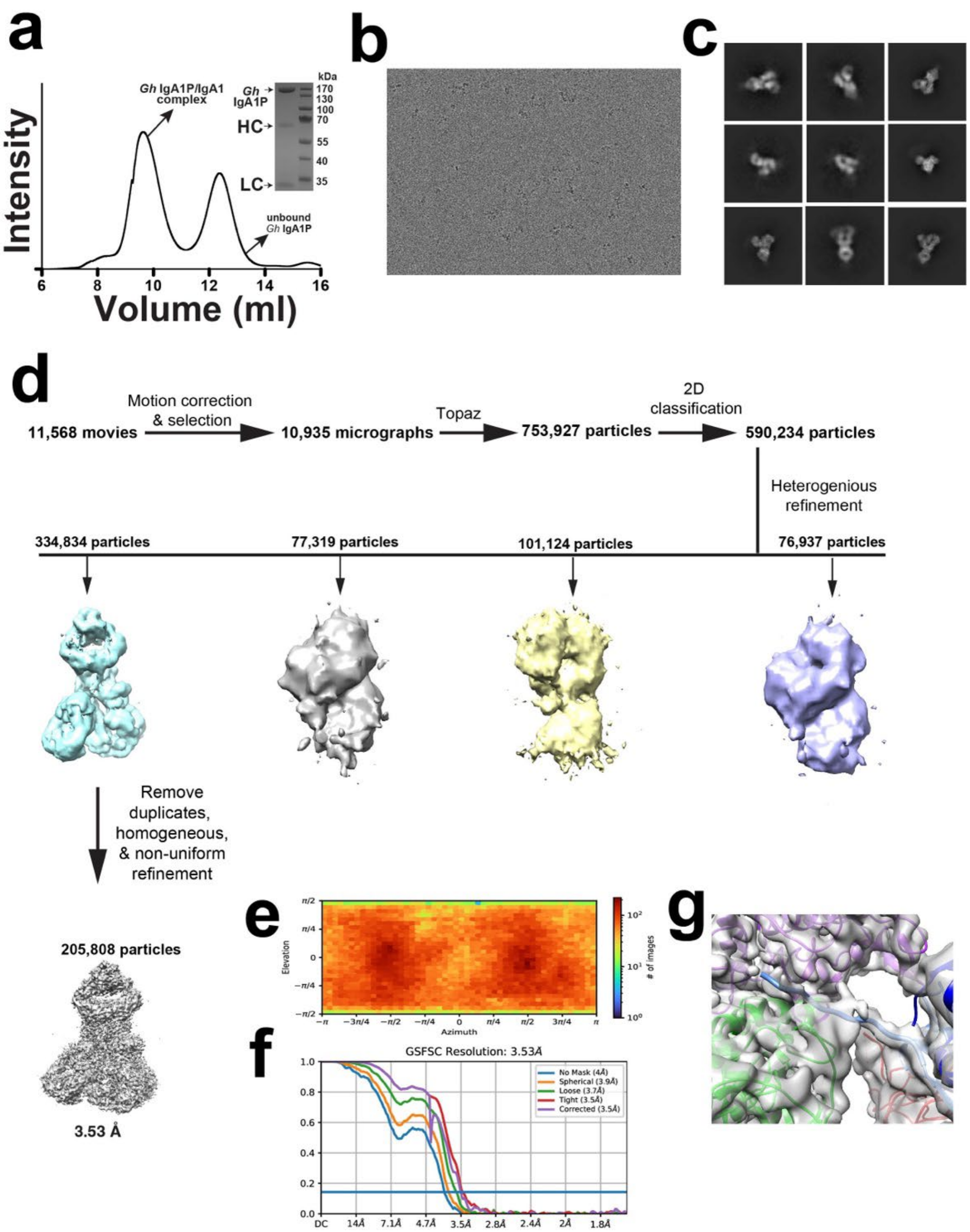

Supplementary Figure 5. Purification and workflow for cryo-EM data processing for substrate bound *G. haemolysans* IgA1P in complex with IgA1. a) Analytical size exclusion chromatography to assess purity of the *G. haemolysans* IgA1P residues 907-2201 complex with IgA1 together with an SDS-PAGE gel analysis of the elution peak indicating the presence of IgA1P, IgA1-HC, and IgA1-LC. b) Raw cryo-EM micrograph c) Representative 2D class averages of substrate-free *G. haemolysans* IgA1P residues 907-2201. d) EM data processing workflow. e) Angular distribution of the final reconstruction. f) Local resolution map of the final density. g) Density and corresponding model for the active site within the complex.
